# Atazanavir-induced unconjugated hyperbilirubinemia prevents vascular hyporeactivity during experimental human endotoxemia

**DOI:** 10.3389/fimmu.2023.1176775

**Published:** 2023-05-16

**Authors:** Mirrin J. Dorresteijn, Douwe Dekker, Jelle Zwaag, Suzanne Heemskerk, Hennie M.J. Roelofs, Paul Smits, Johannes G. van der Hoeven, Frank A.D.T.G. Wagener, Peter Pickkers

**Affiliations:** ^1^Department of Intensive Care Medicine, Radboudumc Center for Infectious Diseases, Radboud University Medical Center, Nijmegen, Netherlands; ^2^Department of Pharmacology and Toxicology, Research Institute for Medical Innovation, Radboud University Medical Center, Nijmegen, Netherlands; ^3^Department of Gastroenterology, Radboud University Medical Center, Nijmegen, Netherlands; ^4^Dentistry-Orthodontics and Craniofacial Biology, Research Institute for Medical Innovation, Radboud University Medical Center, Nijmegen, Netherlands

**Keywords:** antioxidant, heme oxygenase system, atherosclerosis, bilirubin, inflammation, endothelial dysfunction

## Abstract

**Objective:**

Inflammation-induced free radical release is important in the pathogenesis of several diseases, including atherosclerosis and sepsis. Heme oxygenase (HO) breaks down heme into carbon monoxide, iron, and biliverdin. Biliverdin IXα is directly converted to bilirubin by biliverdin reductase. Unconjugated bilirubin is a powerful antioxidant, and elevated levels have beneficial effects in preclinical models and human cardiovascular disease. However, its impact during acute inflammation in humans is unknown. In the present study, we investigated the impact of atazanavir-induced (unconjugated) hyperbilirubinemia on antioxidant capacity, inflammation, and vascular dysfunction in human experimental endotoxemia.

**Approach and results:**

Following double-blinded four-day treatment with atazanavir 2dd300 mg (or placebo), twenty healthy male volunteers received 2 ng/kg *Escherichia coli* lipopolysaccharide intravenously. Blood was drawn to determine the bilirubin levels, antioxidant capacity, and cytokine response. It was demonstrated that following atazanavir treatment, total bilirubin concentrations increased to maximum values of 4.67 (95%CI 3.91-5.59) compared to 0.82 (95%CI 0.64-1.07) mg/dL in the control group (p<0.01). Furthermore, the anti-oxidant capacity, as measured by the ferric-reducing ability of plasma (FRAP), was significantly increased with 36% in hyperbilirubinemia subjects (p<0.0001), and FRAP concentrations correlated strongly to bilirubin concentrations (R2 = 0.77, p<0.001). Hyperbilirubinemia attenuated the release of interleukin-10 from 377 (95%CI 233-609) to 219 (95%CI 152-318) pg/mL (p=0.01), whereas the release of pro-inflammatory cytokines remained unaltered. *In vitro*, in the absence of hyperbilirubinemia, atazanavir did not influence lipopolysaccharide-induced cytokine release in a whole blood assay. Vascular function was assessed using forearm venous occlusion plethysmography after intra-arterial infusion of acetylcholine and nitroglycerin. Hyperbilirubinemia completely prevented the LPS-associated blunted vascular response to acetylcholine and nitroglycerin.

**Conclusions:**

Atazanavir-induced hyperbilirubinemia increases antioxidant capacity, attenuates interleukin-10 release, and prevents vascular hyporesponsiveness during human systemic inflammation elicited by experimental endotoxemia.

**Clinical trial registration:**

http://clinicaltrials.gov, identifier NCT00916448.

## Introduction

Inflammation is the common denominator for numerous diseases, and among them are the most common causes of death in the developed world, including ischemic heart disease, stroke (1) and sepsis (2) In cardiovascular diseases, chronic inflammation, oxidative stress, and endothelial dysfunction contribute to the formation and progression of atherosclerosis (3). Acute inflammatory disorders such as the systemic inflammatory response after trauma, major surgery, or during sepsis are believed to be driven by the same factors: activation of innate immunity and oxidative stress, leading to vascular dysfunction, organ dysfunction, and, ultimately, death (4, 5). Therefore, cardiovascular and infectious diseases research has demonstrated overlap in recent years, focusing on cytoprotective enzyme networks and their anti-inflammatory and anti-oxidative effects (6, 7). The heme oxygenase system is one of the most prominent antioxidant enzymes (8). Heme oxygenase (HO) breaks down heme into carbon monoxide, iron, and biliverdin. Biliverdin IXα is subsequently converted by biliverdin reductase to bilirubin (9).

While historically considered a toxic waste product, bilirubin is the body’s most potent endogenous anti-oxidant (10). Bilirubin is known to inhibit nicotinamide adenine dinucleotide phosphate (NADPH) oxidase (11, 12) and lipid peroxidation, both considered crucial for the development of atherosclerosis (13, 14). Furthermore, it is a known scavenger of reactive nitrogen species (RNS) such as nitric oxide (NO) and peroxynitrite (15), both considered central players in the development of mitochondrial dysfunction in septic shock (13, 16). Indeed, low bilirubin levels are linked to an increased risk for cardiovascular and fibrotic disease (17–21).

On the other hand, subjects with the common Gilbert syndrome, known for a mild lifelong increase in bilirubin concentrations, demonstrate increased antioxidant capacity (22–24), and better endothelium-dependent vasodilation (25). In addition, they are protected from myocardial infarction and peripheral artery diseases in most (23, 25–27) but not all studies (28, 29). Animal experiments have also demonstrated the beneficial effects of bilirubin (or biliverdin) administration in endotoxemia, a model for acute inflammatory disorders such as sepsis (30–32). Data from almost 85 years ago suggested already potent anti-inflammatory effects of bilirubin infusion on arthritis in humans (33). Moreover, beneficial effects on endothelial function were demonstrated in diabetic patients treated with the bilirubin-increasing drug atazanavir (34). Atazanavir, a protease inhibitor used in treating human immunodeficiency virus (HIV) infection, is known to inhibit uridine diphosphate-5’-glucuronosyltransferase A1 (UGT1A1), thereby creating a pharmacologically-induced Gilbert-syndrome. We used this effect to create hyperbilirubinemia in the healthy volunteers participating in the present study. We modified the dosing regime in agreement with a previous study to produce sufficient bilirubin (34). The human endotoxemia model is known for inducing a predictable inflammatory response and vascular hyporesponsiveness. Therefore, administering lipopolysaccharide (LPS) to healthy volunteers enables the investigation of possible therapeutical targets in inflammatory disorders (35, 36). In the present study, we aimed to investigate the effects of atazanavir-induced mild hyperbilirubinemia on anti-oxidant capacity, systemic inflammation, and vascular hyporeactivity elicited by experimental human endotoxemia.

## Materials and methods

All study procedures were approved by the local ethical committee and in accordance with the declaration of Helsinki. This study was registered at www.clinicaltrials.gov under the number NCT00916448.

### Subjects

All participating subjects gave written informed consent prior to inclusion. A screening visit within two weeks of the experiments demonstrated no abnormalities in medical history or physical examination. Routine laboratory tests and an electrocardiogram (ECG) showed no abnormalities. All subjects tested negative for HIV and Hepatitis B. Total bilirubin levels below 0.88 mg/dL (below 15 μmol/L) were obligatory to exclude subjects with Gilbert syndrome. All subjects were non-smokers with no signs of previous hypertension or cardiovascular disease. Furthermore, no use of (over the counter or prescribed) drugs was allowed within two weeks before enrollment until the end of the study. Ten h before the infusion of LPS, subjects refrained from consuming alcohol, caffeine, and food.

### Study design

In clinical practice, HIV-patients are treated with 300 mg of atazanavir once daily boosted with 100 mg of ritonavir. A study performed by Acosta et al. demonstrated that when atazanavir is used in a 300 mg twice daily dose, it is well tolerated and leads to a lower Cmax and AUC than atazanavir 300 mg once daily boosted with ritonavir (37). However, subjects in the twice-daily atazanavir group did experience hyperbilirubinemia. A side effect that we chose to use as a therapeutic agent. Our research group has previously used this 600mg dosage of atazanavir successfully to increase bilirubin levels in diabetes patients (34). In this randomized placebo-controlled parallel study, subjects were treated with atazanavir 300 mg or placebo twice daily for four consecutive days before the LPS infusion in a double-blinded fashion.

Randomization was performed by an independent research nurse using sealed envelopes. Per the inclusion criterion, all subjects started treatment with bilirubin levels below 0.88 mg/dL. After two days into the treatment protocol, bilirubin levels were checked by the independent research nurse to ensure that total concentrations would not exceed desired values. Given the double-blind, placebo-controlled nature of the study, investigators were not informed about bilirubin concentrations. However, when bilirubin concentrations exceeded 4.68 mg/dL (equal to 80 μmol/L) the subject was excluded from further participation by the research nurse. Excluded subjects were replaced in the study protocol without unblinding the involved investigators. After 4 days of treatment, subjects were admitted to the research-intensive care unit of the Radboud university medical center. An additional ECG was performed to exclude conduction disorder as a side effect of atazanavir.

**Figure 1:**
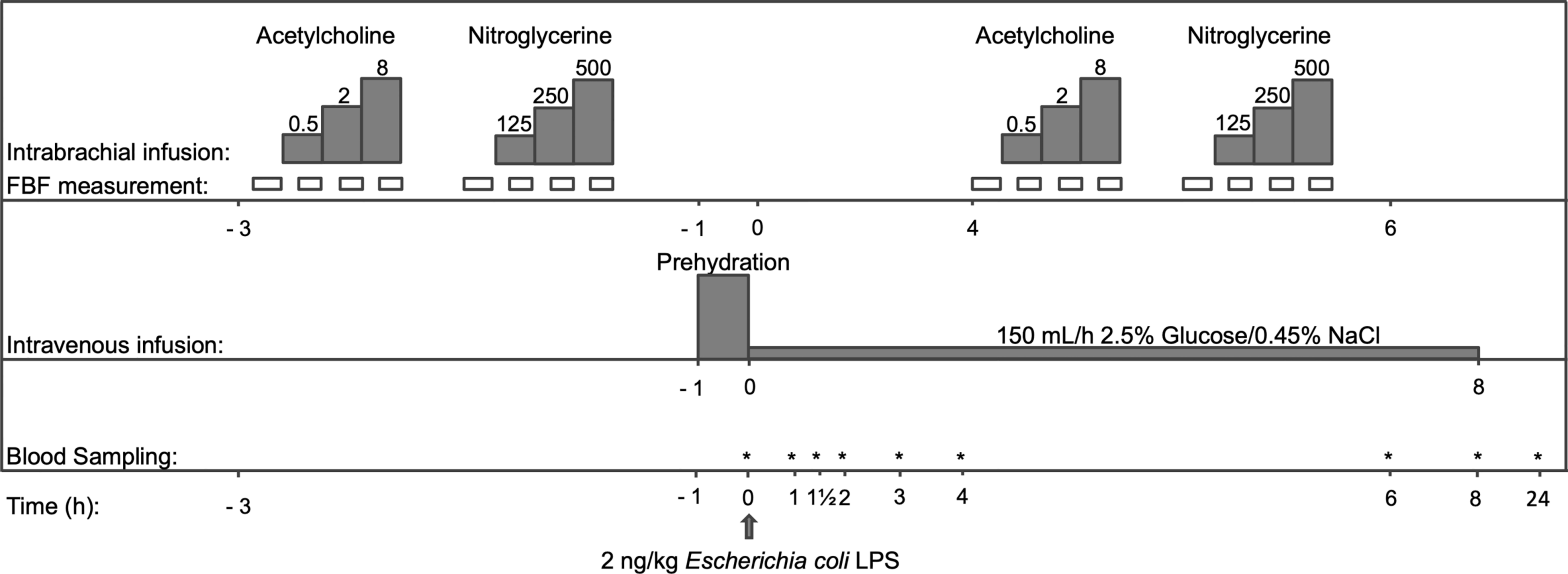
Flowchart of the study protocol. After 4 days of taking either atazanavir (n=10) or placebo (n=10), but prior to LPS infusion at 0 h, dose-response curves to acetylcholine and nitroglycerine were determined. Thereafter, prehydration (1,5L of 2,5% Glucose/0.45% NaCl) was infused and blood was sampled before LPS infusion and serially after that. At 4 h after LPS, dose-response curves to acetylcholine and nitroglycerine were repeated. FBF indicates forearm blood flow. The dosing of acetylcholine was 0.5-2-8 μg/min/dL forearm volume; nitroglycerin was dosed at 125-250-500 ng/min/dL forearm volume. *: time points that blood samples were taken.

After local anesthesia (lidocaine 20 mg/mL), the brachial artery was cannulated with a 20-gauge catheter connected to an arterial pressure monitoring line (Edwards Lifesciences LLC, Irvine CA, USA connected to a Philips IntelliVue MP70 monitor, Philips Medical Systems, Eindhoven, The Netherlands) to enable the administration of vasodilating drugs, blood sampling and the continuous monitoring of blood pressure. A second cannula was placed in an antecubital vein to permit infusion of prehydration fluid (1.5 L of 2.5% glucose/0.45% saline solution), LPS, and the continuous infusion of 150 mL/h 2.5% glucose/0.45% saline. After determination of the baseline responses to acetylcholine and nitroglycerin, purified LPS (US Standard Reference Endotoxin *E. coli* O:113) obtained from the Pharmaceutical Development Section of the National Institutes of Health (Bethesda, MD, USA) was administered at a dose of 2 ng/kg body weight. Blood was sampled serially, and body temperature was determined every 30 minutes (min)(Firsttemp Genius 2, Covidien, Dublin, Ireland) (**Figure 1**).

### Laboratory tests

All blood samples were drawn from the arterial line, except for samples obtained at t=24 hours (h) when blood was sampled using regular vena puncture. Bilirubin was determined before and every two h after LPS infusion in lithium heparin plasma using immunologic detection according to the manufacturer’s instructions (Aeroset, Abbott Laboratories, Abbott Park, IL, USA). The ferric-reducing ability of plasma (FRAP) assay was performed in duplicate as described previously (38). Ferric reducing ability of plasma values were obtained using a 7-point calibration curve of known amounts of Fe2+ and expressed in mmol Fe2+/L. The concentrations of circulating cytokines tumor necrosis factor (TNF)-α, interleukin (IL)-6, IL-8, IL-10 and monocyte chemotactic protein (MCP)-1, as well as concentrations of endocan, endothelial (E) and platelet (P)-selectins and soluble vascular cell and inter-cellular adhesion molecules (VCAM-1 and ICAM-1) were determined in EDTA or lithium heparin plasma obtained immediately before LPS administration and serially thereafter (see **Figure 1** for details). Measurements of all cytokines and the soluble adhesion molecules VCAM-1 and ICAM-1 were performed by a multiplex assay according to the manufacturer’s instructions (Bio-plex cytokine assay, BioRad, Hercules, CA, USA at Luminex 100, Luminex Corporation, The Netherlands). The soluble adhesion molecules E-and P-selectin were determined by Enzyme-Linked Immuno Sorbent Assay (ELISA) (R&D systems, Minneapolis, USA). Endocan was measured using a sandwich ELISA (Lunginnov, Lille, France).

C-reactive protein (CRP) levels were measured just before and at 8 and 24 h after LPS in lithium heparin plasma (Aeroset, Abbott Laboratories, Abbott Park, IL, USA). Leukocyte counts were determined in EDTA anti-coagulated blood using flow-cytometry (Sysmex XE-2100, Goffin Meyvis, Etten-Leur, The Netherlands) just before and serially after LPS infusion.

### *In vitro* LPS stimulation

To exclude a direct pro- or anti-inflammatory effect of atazanavir unrelated to its induction of hyperbilirubinemia, an *in vitro* experiment incubating whole blood in the presence or absence of atazanavir and/or LPS was performed. An atazanavir stock solution of 1 mg/mL was prepared as previously described (39) and dissolved in DMSO to prevent sedimentation. Further dissolution was performed in RPMI 1640 (Sigma-Aldrich, Zwijndrecht, The Netherlands) to reach a final concentration of dimethyl sulfoxide (DMSO) of 0.1%. Whole blood of 6 healthy volunteers was incubated with atazanavir 0, 1, 5, or 10 μmol/L and LPS 0, 1, 10 ng/mL for 24 h at 37°C under 5% CO2. Thereafter, the incubate was centrifuged at 20.000 g for 5 min. Plasma was aspirated and an ELISA was performed for TNF-α and IL-10 (R&D systems, Minneapolis, USA).

### Forearm blood flow measurements

Forearm blood flow (FBF) was determined using venous occlusion plethysmography (Filtrass, Domed, Munich, Germany) as previously described (40, 41). Briefly, venous occlusion was achieved by inflating the upper arm cuff to 45 mmHg. Strain gauges were placed on the forearm and connected to plethysmographs to measure changes in forearm volume in response to inflation of the venous-congesting cuffs. FBF and drug administration were normalized to forearm volume as measured with the water displacement method and expressed in milliliters (mL) per min per deciliter (dL) forearm volume (mL/min/dL). After instrumentation, a resting period of 30 min was kept ensuring stable FBF. After that, a dose-response curve to intrabrachial infusion of acetylcholine (Miochol,Thea Pharma NV, Zoetermeer, the Netherlands) and nitroglycerine (Nitropohl, Pohl-Boskamp, Hoofddorp, The Netherlands) was determined. Baseline FBF was determined for five min, after which increasing doses of acetylcholine at 0.5, 2, and 8 μg/min/dL forearm volume were infused in equal volumes. Each dose was administered for 5 min, while measurements of FBF were performed in the last 3 min of each dose. After a washout period of 15 min and after another baseline measurement, nitroglycerin was infused in the same fashion at doses of 125, 250, and 500 ng/min/dL.

Since infusion of LPS is known to induce vascular hyporeactivity to vasodilators after 4 h and until 6 h after administration (42, 43), we repeated these measurements at t=4 h to determine the effects of atazanavir-induced hyperbilirubinemia on LPS-induced vascular hyporeactivity.

### Data analysis and statistics

Values are expressed as geometric mean (95% confidence interval (CI)) unless described otherwise. Because of the small sample size, log transformation was performed to ensure a Gaussian distribution. A two-way repeated-measures analysis of variance (ANOVA) was used to test variation over time, the variation between interventions, and the interaction between time and intervention (IBM SPSS statistics 20 software, Amsterdam, The Netherlands). One-way ANOVA (GraphPad Prism version 5.03 for Windows, GraphPad Software, San Diego California, USA) analyzed changes over time alone. We used Student’s t-test (Graphpad) on log-transformed data to compare differences in baseline parameters between groups and between baseline and peak values. A p*-*value<0.05 was considered to indicate significance. Given this study’s explorative “proof-of-concept” nature, no formal sample size calculation was performed, and no subgroup analyses were conducted.

## Results

### Demographic data and safety

Baseline characteristics are presented in **Table 1**. There were no significant differences between the groups. The study drugs were well tolerated, and the subjects reported no side effects.

**Table 1:** Baseline characteristics.

	Placebo (n=10)	Atazanavir (n=10)	p-value
**Height, cm**	182 (179-185)	182 (177-187)	0.9
**Weight, kg**	73.7 (68.4-79.4)	76.7 (70.4-83.4)	0.5
**Body mass index, kg/m^2^**	22.3 (20.8-23.9)	23.2 (21.4-25.1)	0.3
**Age, years**	23.0 (21.4-24.7)	21.7 (19.8-23.9)	0.3
**Heart rate, bpm**	60 (54-66)	63 (57-69)	0.5
**Systolic blood pressure, mmHg**	121 (112-130)	125 (119-131)	0.5
**Diastolic blood pressure, mmHg**	74 (70-78)	77 (71-82)	0.4
**Forearm Volume, mL**	1401 (1324-1482)	1415 (1292-1549)	0.9
**Total cholesterol, mmol/L**	3.3 (2.8-3.7)	3.2 (2.8-3.6)	0.7
**LDL cholesterol, mmol/L**	1.9 (1.5-2.3)	1.7 (1.3-2.1)	0.4
**HDL cholesterol, mmol/L**	0.97 (0.85-1.1)	1.00 (0.88-1.12)	0.7
**Non-HDL cholesterol, mmol/L**	2.3 (1.9-2.8)	2.1 (1.7-2.6)	0.6
**Triglycerides, mmol/L**	0.8 (0.6-1.1)	1.0 (0.8-1.2)	0.3

All data are expressed as geometric mean (95% confidence interval). P-values were obtained using an unpaired Student’s t-test on log-transformed data to ensure normal distribution. No significant differences between groups were observed.

A total of 30 subjects were included in the study, 10 could not complete the study protocol for different reasons: five subjects were excluded because of a too steep rise in bilirubin following intake of study medication, as measured two days before the planned LPS administration. Two subjects experienced a vagal response during the placement of the cannulas, 1 subject was unable to plan the experiment, 1 subject made errors in medication intake, and 1 subject had a new 1^st^ degree atrioventricular block on the morning of the experiment. All subjects excluded after starting the study medication were replaced to ensure the equal distribution of subjects in the two treatment arms.

### Bilirubin and anti-oxidant capacity

Before the start of atazanavir/placebo treatment, bilirubin levels averaged 0.54 (95%CI: 0.49-0.58) mg/dL in subjects using atazanavir versus 0.57 (95%CI: 0.50-0.65) mg/dL in the placebo group (p=0.5). After 4 days of treatment, bilirubin averaged 3.08 (95%CI: 2.62-3.61) mg/dL in subjects treated with atazanavir versus 0.48 (95%CI: 0.37-0.52) mg/dL in subjects treated with placebo (p<0.01).

**Figure 2:**
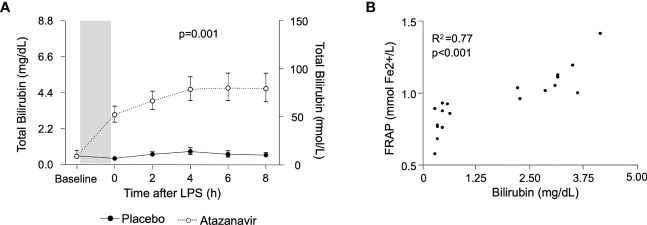
**(A)** Bilirubin concentrations in subjects treated with placebo (•) and atazanavir (∘). The baseline depicts values before the start of treatment with a placebo or atazanavir. The shaded area represents this 4-day treatment window. Afterwards, measurements were performed before (0h) and serially after administering 2 ng/kg *Escherichia coli* LPS. Data are represented as geometric mean and 95% confidence interval. P-value was obtained by repeated measures ANOVA on log-transformed data of the complete curve. **(B)** The correlation between bilirubin and FRAP. Values were obtained just before administering 2 ng/kg *Escherichia coli* LPS. Correlations were determined using Pearson’s correlation coefficient on log-transformed data.

During experimental endotoxemia, a further increase in bilirubin was observed in both groups. In subjects treated with atazanavir, bilirubin levels peaked after 6 h with maximum concentrations of 4.67 (95%CI: 3.91-5.59) mg/dL while in the placebo group, maximum values of 0.82 (95%CI: 0.64-1.07) were reached at 4 h after LPS infusion (over time both p<0.001, between groups p=0.001) (**Figure 2A**).

The total antioxidant capacity was assessed by measurement of FRAP. It was significantly increased in subjects treated with atazanavir averaging 1.09 (95%CI: 1.01-1.18) mmol Fe^2+^/L compared to 0.80 (95%CI: 0.72-0.89) mmol Fe^2+^/L in subjects treated with placebo (p<0.0001) and did not change following LPS administration (over time p=0.5 in both the atazanavir group and the placebo group). Furthermore, there was a strong correlation between bilirubin and FRAP concentrations (Spearman R^2^: 0.77, p<0.001) (**Figure 2B**).

### Inflammatory effects *in vivo* and *in vitro*

**Figure 3:**
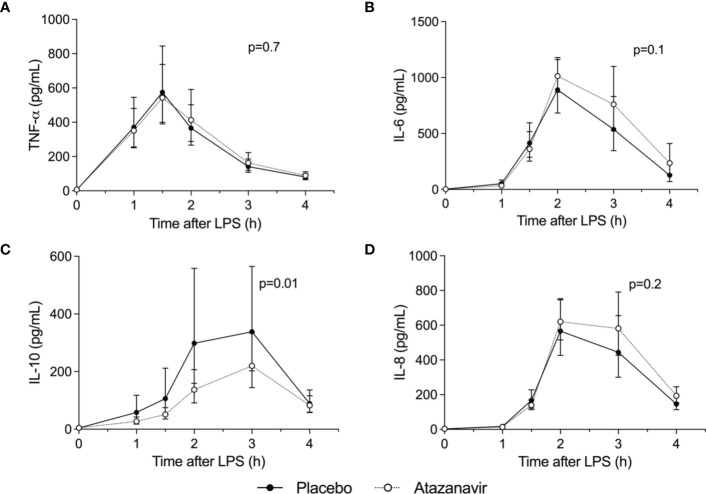
Cytokine concentrations. Levels were determined in the absence (n=10 depicted as •) and presence (n=10, depicted as ∘) of atazanavir after administration of 2 ng/kg *Escherichia coli* LPS to healthy volunteers at 0 h. Data are presented as geometric mean and 95% confidence interval. P-values were obtained using repeated measures ANOVA on log-transformed data of the complete curve. **(A)** TNF-α: tumor necrosis factor-α, **(B)** IL-6: interleukin-6, **(C)** IL-10: interleukin-10, **(D)** IL-8: interleukin-8.

As shown in **Figure 3**, LPS infusion increased all measured cytokines. Maximum concentrations for the pro-inflammatory cytokine TNF-α were 578 (95%CI: 443-753) pg/mL in subjects treated with atazanavir versus 580 (95%CI: 395-852) pg/mL in subjects treated with placebo (p-value over time both <0.001, between groups p=0.7). Concentrations of IL-6 peaked at 1082 (95%CI: 914-1280) pg/mL in hyperbilirubinemia subjects compared to 890 (95%CI: 683-1160) pg/mL in the placebo group (over time both p<0.001, between groups p=0.1). IL-8 reached maximum values of 693 (95%CI: 583-825) pg/mL in subjects treated with atazanavir versus 590 (95%CI: 444-784) pg/mL in subjects treated with placebo (over time p<0.001, between groups p=0.2).

**Figure 4:**
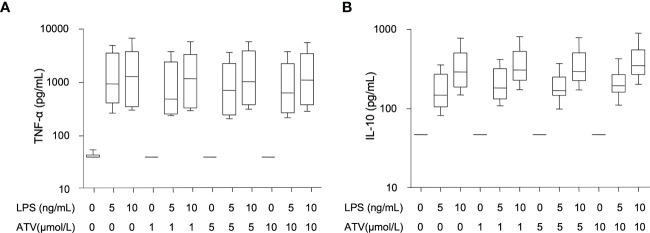
*In vitro* effects of atazanavir and LPS. **(A)** Concentration of tumor necrosis factor-α (TNF-α) 24 hrs after incubation of whole blood with rising doses of *Escherichia coli* LPS and/or atazanavir (ATV) (n=6). Both LPS doses significantly increased TNF-α concentrations (p<0.001), while increasing amounts of atazanavir did not affect TNF-α concentrations. **(B)** Concentrations of interleukin (IL)-10 after 24 hrs of whole blood incubation (n=6) with rising doses of *Escherichia coli* LPS and/or atazanavir (ATV). Both doses of LPS significantly increased IL-10 concentrations (p<0.001), while increasing doses of atazanavir did not affect IL-10. Whiskers are depicted as minimum and maximum values. P-values were obtained using Student’s *t-*test on log-transformed data.

The endotoxemia-induced increase in anti-inflammatory cytokine IL-10 was attenuated in subjects treated with atazanavir: 219 (95%CI: 152-318) pg/mL versus 377 (95%CI: 233-609) pg/mL in subjects in the placebo group (over time, both p<0.001, between groups p=0.01). (**Figure 4**) Moreover, peak levels of IL-10 were correlated to peak levels of bilirubin (Pearson R^2^= -0.19, p=0.05) and area under the curve for bilirubin (Spearman R^2^: -0.25, p=0.03).

MCP-1 peaked at 5828 (95%CI: 4183-8063)pg/mL in atazanavir-treated subjects while subjects treated with placebo had maximum values of 6835 (95%CI: 5224-8943) pg/mL (over time p<0.001, between groups p= 0.6, data not shown).

CRP was maximally increased at 24 h following LPS infusion but did not differ between groups (average concentration of 39 (95%CI: 33-47) mg/L in the atazanavir-group compared to 35 (95%CI: 29-44) mg/L in the placebo group, p=0.3).

As expected, in our *in vitro* experiments, increasing doses of LPS induced increasing concentrations of TNF-α and IL-10. (**Figure 4**) However, increasing concentrations of atazanavir did not modulate the release of TNF-α or IL-10 during whole blood incubation for 24 h nor did atazanavir influence the release of TNF-α or IL-10 with increasing doses of LPS.

### Effects on systemic hemodynamic response

LPS caused a significant decrease in mean arterial pressure (MAP) and increase in heart rate (P<0.001). Treatment with atazanavir or placebo did not influence endotoxemia-induced changes in hemodynamics. At 4 h after LPS infusion, MAP was decreased by 20% in subjects receiving atazanavir versus a decrease of 16% in subjects receiving placebo (**Table 2**, over time both groups p<0.001, no significant difference between groups). Heart rate increased by 30% in hyperbilirubinemic subjects versus 31% in the placebo group. (**Table 2**, over time both groups p<0.001, no significant difference between groups).

**Table 2:** Hemodynamic response, temperature, and forearm blood flow after 2 ng/kg *E*. *coli* LPS infusion.

		Before LPS	T=4 h	p-value over time	p-value between groups
**Heart Rate, bpm**	Placebo Atazanavir	70 (62-79) 73 (65-81)	92 (85-100) 95 (87-104)	<0.001 <0.001	0.3
**MAP, mmHg**	Placebo Atazanavir	93 (87-100) 96 (91-102)	78 (73-84) 77 (72-82)	<0.001 <0.001	0.6
**Temperature, °C**	Placebo Atazanavir	36.8 (36.7-37.0) 36.8 (36.6-37.1)	37.8 (37.4-38.3) 38.0 (37.2-38.9)	<0.001 <0.001	0.6
**FBF, mL/min/dL**	Placebo Atazanavir	3.2 (2.1-4.8) 4.5 (3.0-6.7)	8.1 (6.5-10.1) 6.9 (4.5-10.5)	<0.001 0.2	NS*

MAP, mean arterial blood pressure; FBF, forearm blood flow. Data are expressed as geometric mean and 95% confidence interval. P-values were all calculated on log-transformed data. Values over time were obtained using one-way ANOVA while p-values between groups were obtained using repeated measures analysis of variance or, in the case of forearm blood flow, using an unpaired Student’s t-test. *NS is used to indicate the non-significant differences between forearm blood flow in subjects using placebo or atazanavir before LPS (p=0.2) and after LPS (p=0.5).

### Effects on endothelial dysfunction and activation

**Figure 5:**
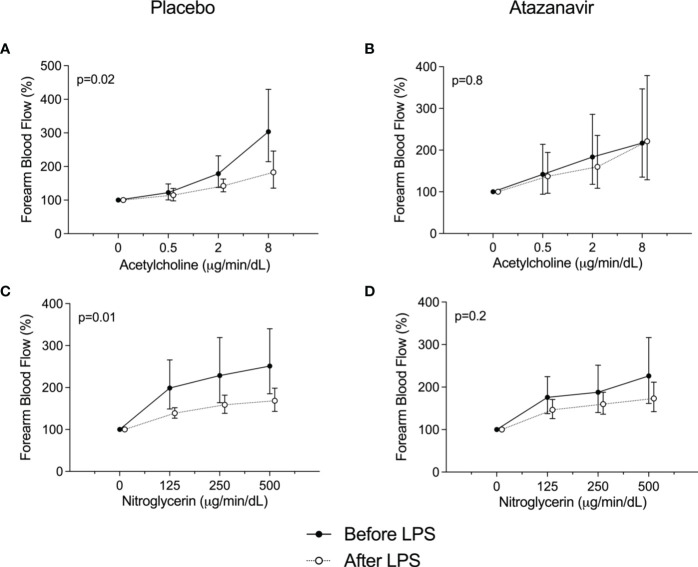
Forearm blood flow dose-response curves. **(A)** Responses to acetylcholine before (•) and 4 h after the administration of 2 ng/kg *Escherichia coli* LPS (∘) to healthy subjects using placebo. **(B)** Responses to acetylcholine before (•) and 4 h after the administration of 2 ng/kg *Escherichia coli* LPS (∘) to healthy subjects using atazanavir. **(C)** Responses to nitroglycerin before (•) and 4 h after the administration of 2 ng/kg *Escherichia coli* LPS (∘) to healthy subjects using placebo. **(D)** Responses to nitroglycerin before (•) and 4 h after the administration of 2 ng/kg *Escherichia coli* LPS (∘) to healthy subjects using atazanavir. Data are presented as % compared to baseline and expressed as geometric mean and 95% confidence interval. P-values were obtained using repeated measures ANOVA on log-transformed data of the complete curve.

FBF assessed by venous occlusion plethysmography increased in both groups after LPS infusion as demonstrated in **Table 2**. In subjects treated with placebo and before the administration of LPS, the highest dose of acetylcholine (8 µg/min/dL forearm tissue) increased flow to 304 (95%CI: 214-430)% compared to baseline. At 4 h after LPS infusion the response was attenuated to a maximum response of 183 (95%CI: 136-246)% compared to the baseline measured at 4 h. (p=0.02, **Figure 5A**).

The attenuated vasodilatory response following LPS infusion was not observed in subjects treated with atazanavir. After the highest dose of acetylcholine and before the administration of LPS, flow increased to 221 (95%CI: 129-378)% compared to baseline, whereas 4 h after LPS administration flow increased to 217 (95%CI: 135-347)% after 8 µg/min/dL acetylcholine.(p=0.8, **Figure 5B**).

For nitroglycerine, similar effects were found. In subjects treated with placebo, nitroglycerine at a dose of 500 ng/min/dL increased flow to maximum values of 251 (95%CI: 185-340)% compared to baseline, whereas at 4 h after LPS the maximum response was 169 (95%CI: 143-198)% (p=0.01). In hyperbilirubinemic subjects, the attenuated response following LPS infusion was not observed: 226 (95%CI: 162-316) % compared to baseline before LPS and 173 (95%CI: 142-211)% after LPS infusion. (p=0.2, **Figures 5C, D**).

**Figure 6:**
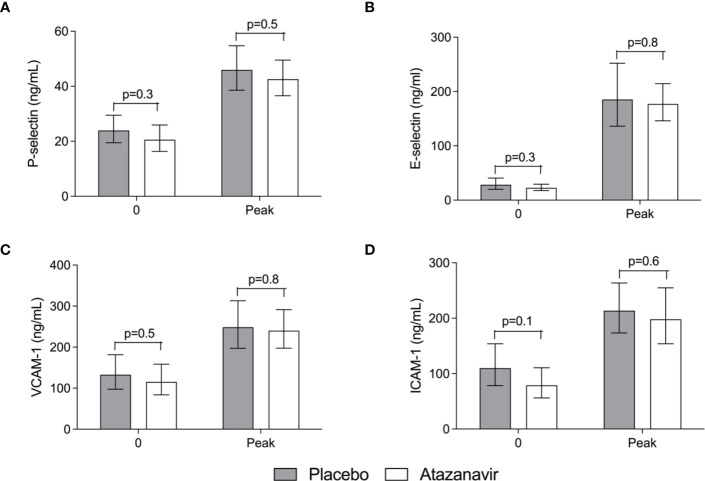
Soluble endothelial adhesion molecules in the serum. **(A)** Soluble Platelet (P)-selectin. **(B)** Soluble Endothelial (E)-selectin. **(C)** Soluble Vascular cell adhesion molecule (VCAM)-1. **(D)** Soluble Intercellular adhesion molecule (ICAM)-1. Values obtained before and at the peak after administration of 2 ng/kg *Escherichia coli* LPS to healthy volunteers in the absence (grey bars) and presence (white bars) of atazanavir. In both treatment arms, LPS induced an increase for all molecules (p<0.01). Data are presented as geometric means and a 95% confidence interval. P-values between groups were obtained using an unpaired Student’s *t*-test on log-transformed data.

Following LPS administration, the soluble endothelial adhesion molecules E-selectin, P-selectin, VCAM-1, and ICAM-1 significantly increased over time (p<0.01). No significant differences could be demonstrated between subjects treated with atazanavir or placebo. (**Figure 6**) Furthermore, the circulating proteoglycan endocan was increased from 2.7 (2.3-3.1) at baseline to 6.7 (5.9-7.7) ng/mL at 3 h after LPS in hyperbilirubinemic subjects compared to an increase from 2.7 (2.1-3.3) to maximum values of 7.3 (5.6-9.6) at 3 h after LPS infusion in placebo-treated subjects (over time both p<0.001). No significant differences between groups were observed.

## Discussion

The present study demonstrates that atazanavir-induced hyperbilirubinemia indeed increases anti-oxidative capacity. Moreover, vascular hyporeactivity during experimental endotoxemia was prevented in subjects with atazanavir-induced hyperbilirubinemia. Surprisingly, endotoxemia-induced IL-10 release was attenuated following atazanavir-induced hyperbilirubinemia.

Our data indicate that bilirubin levels in this range (3.08 (95%CI: 2.62-3.61) mg/dL) exert relevant effects on oxidative stress, inflammation, and vascular reactivity in humans *in vivo*. In the human body, bilirubin is present in three forms: the majority is albumin-bound unconjugated bilirubin, a very small fraction is free unconjugated bilirubin, and the remainder is conjugated bilirubin. By using atazanavir, the main form of the increased bilirubin levels will be albumin-bound and unconjugated. Its anti-oxidative effects in this form include inhibition of NADPH oxidase (11, 12), reduced lipid-peroxidation, scavenging of peroxyl radicals, and RNS, thereby protecting fatty acids and decreasing the oxidative capacities of hydroxyl, superoxide anion radicals, and peroxynitrite (extensively described in a review by Stocker (10)). Besides beneficial effects in cardiovascular diseases by preventing the formation and progression of atherosclerosis and vascular dysfunction, these effects could also benefit septic patients since these substances are related to mitochondrial dysfunction, which is important in developing organ dysfunction (13, 16).

Vascular dysfunction is strongly associated with atherosclerosis and cardiovascular disease, and increased oxidative stress is of utmost importance for its development (44). Our data show that LPS-induced vascular hyporeactivity is absent during mild hyperbilirubinemia. We hypothesize that the anti-oxidative capacities of bilirubin mediate this effect. Similar effects have been demonstrated for the anti-oxidant vitamin C. Both locally and systemically administered vitamin C was associated with an improved endothelial function using the same experimental human endotoxemia model (45, 46). Moreover, it is known that healthy subjects with mild hyperbilirubinemia have significantly enhanced endothelium-dependent vasodilation and flow-mediated dilation compared to subjects with normal or low bilirubin (25, 47). Of interest, similar ameliorating effects on endothelium-dependent vasodilation were demonstrated in type 2 diabetes patients with atazanavir-induced hyperbilirubinemia (34). These data indicate that modulation of oxidative and nitrosative stress may be of clinical relevance in different groups of patients.

The attenuation of the response to the non-endothelium-dependent vasodilator nitroglycerin observed in our experiments was not present in some previous studies (43, 45). By contrast, two other studies demonstrated a similarly clear and significant attenuation of the response to sodium nitroprusside or nitroglycerin during experimental endotoxemia (48, 49). One could speculate that inducible nitric oxide synthase (iNOS)-derived nitric oxide (NO) after endotoxin administration induces a mild tolerance to NO-donors such as nitroglycerin. Since bilirubin is a known iNOS inhibitor (12, 32), it is tempting to speculate that this may explain why the effect is absent in subjects treated with atazanavir-induced hyperbilirubinemia.

The beneficial effects of elevated bilirubin levels on inflammatory markers were demonstrated in various inflammation models. In some studies, TNF-α secretion was attenuated (30, 32), while no effects on cytokine concentrations were observed in another study (11). Nevertheless, pretreatment with bilirubin before LPS administration reduces symptoms, attenuates iNOS release and improves survival rates in animal models (30, 32). Of interest, an average (lifelong) concentration of 1.14 mg/dL in a human study population with Gilbert syndrome is known to have protective effects on cardiovascular disease compared to an average of 0.69 mg/dL in control patients (26). These data suggest that relatively small changes in bilirubin concentration could already elicit beneficial effects. Others have used much higher concentrations in rodents with a single bolus that reached peak concentrations of 6 mg/dL (30) We aimed for bilirubin levels around 4 mg/dL based on a previous study from our research group (34). In that study, atazanavir led to a bilirubin concentration of 3.8 mg/dL, demonstrating beneficial effects on endothelial function in type 2 diabetes (34). Intriguingly, an ancient human study infusing bilirubin and bile salts from 1938 showed that levels between 4 and 16 mg/dL attenuated inflammatory signs and pain perception in arthritis patients (33). Again, we cannot exclude that much lower bilirubin levels also evoke anti-inflammatory or vascular effects in human inflammatory disorders.

Our research was intended as a proof of principle study on the effects of bilirubin in acute inflammation. We have demonstrated that it is feasible to increase endogenous bilirubin concentrations in a controlled manner to investigate the effects of increased bilirubin levels in humans. Pharmacologically-induced Gilbert syndrome’ is currently mainly feasible for research purposes. Possible side effects and interactions with the metabolism of other drugs make atazanavir an unfit tool to increase bilirubin levels in patients. Interestingly, we recently demonstrated that bilirubin itself could also be safely infused into humans (50), which could open new possibilities for therapeutical purposes.

In our study, endotoxemia itself induced a modest increase in bilirubin. Several mechanisms may explain this effect. LPS administration results in a downregulation of UGT1A1 through nuclear factor (NF)-κB and, therefore, can slightly increase bilirubin (51). Additionally, TNF-α and IL-6 reduce bilirubin clearance in the liver (52). Furthermore, induction of HO-1 that breaks down heme to form bilirubin (most likely occurring in the tissues rather than in circulating cells (53)) cannot be excluded.

Intriguingly, harnessing against inflammatory and oxidative tissue injury has recently been recognized as more important than just targeting invading pathogens (8, 54). Solely eliminating pathogens may leave the patient at risk of dying from collateral tissue injury (8, 54–57), like vascular and organ dysfunction. Inducing *disease tolerance* by activating antioxidant and anti-inflammatory systems may protect against these injurious tissue insults (55). HO-activity has recently been shown to mediate disease tolerance (58, 59), suggesting that the HO-effector molecule bilirubin could also mediate protection. Since bilirubin shows such potent protective effects in both acute (this study) and chronic inflammation (34), increasing bilirubin to mediate tissue protection warrants further exploration (50). Disease tolerance and harnessing against pathogens-induced tissue injury and vascular dysfunction may be mediated by mild hyperbilirubinemia or induction of HO-activity.

One could argue that the effects found in our study were provoked by atazanavir rather than bilirubin. Protease inhibitors in HIV patients are known for their disadvantageous effects on lipid profiles and their adverse effects on endothelial function (60, 61). Both are related to the increased risk for cardiovascular disease in HIV patients. However, a study on healthy volunteers demonstrated that atazanavir treatment did not influence lipid profiles or significantly impact the response to endothelium-dependent vasodilator methacholine (61). In accordance, we demonstrated that short-term treatment with atazanavir in our study does not affect the response to acetylcholine before LPS administration (data not shown) or lipid profiles indicating that inflammation is the most likely cause for the vascular dysfunction in our study. However, a direct effect of atazanavir or its metabolites on endothelial function or inflammation after LPS cannot be entirely excluded. Nevertheless, additional *in vitro* experiments demonstrated that atazanavir does not influence the release of TNF-α or IL-10 (in the presence or absence of LPS), making it less likely that atazanavir itself interfered with our cytokine results.

The strength of the present study includes the fact that the effects of atazanavir-induced hyperbilirubinemia were investigated in a well-explored, standardized, reproducible model for inflammation, thereby gaining insight into the impact of bilirubin during acute systemic inflammation *in vivo* in humans. Naturally, it is a proof of principle study limited by the number of subjects participating and the preclinical model used. Future studies are warranted to explore the effects of bilirubin in chronic and acute human inflammatory diseases.

In conclusion, the present study is the first to demonstrate that hyperbilirubinemia elicited by atazanavir improves the anti-oxidant status, reduces IL-10 release, and prevents vascular hyporeactivity during experimental endotoxemia in humans. Based on these data, further studies utilizing possible other techniques to increase bilirubin concentrations are warranted to investigate its potential therapeutic applications in patients suffering from inflammatory diseases.

## Data availability statement

The original contributions presented in the study are included in the article/supplementary materials, further inquiries can be directed to the corresponding author/s.

## Ethics statement

All study procedures were approved by the local ethical committee and in accordance with the declaration of Helsinki. This study was registered at www.clinicaltrials.gov under the number NCT00916448. The patients/participants provided their written informed consent to participate in this study.

## Author contributions

Conceptualization: MD, DD, PS, FW, PP. Investigation: MD, DD, JZ, SH, HR. Writing-Review, and Editing: MD, DD, PS, JH, FW, PP. All authors contributed to the article and approved the submitted version.
